# Frail patients having vascular surgery during the early COVID‐19 pandemic experienced high rates of adverse perioperative events and amputation

**DOI:** 10.1111/ans.17810

**Published:** 2022-06-08

**Authors:** Australian and New Zealand Vascular Trial Network (ANZVTN), Sarah J. Aitken, Bernard Allard, Nishath Altaf, Noel Atkinson, Omar Aziz, Ruth Battersby, Ruth Benson, Jennifer L. Chambers, Gabriella Charlton, Chloe Coleman, Joseph A. Dawson, Anastasia Dean, Bedanta S. Dhal, Robert Fitridge, John Gan, Joseph Hanna, Andrew T. Hattam, Martin Hein, Kay Hon, Samantha Khoo, Joseph Kilby, Beatrice Kuang, Kai Wen Leong, Eunice Lim, Ju‐wei N. Liu, David N. McClure, Shreya Mehta, Jana‐Lee Moss, Juanita Muller, Korana Musicki, Sandip Nandhra, Michael J Papanikolas, Fernando Picazo Pineda, Franklin Pond, Nandhini Ravintharan, Toby Richards, Hani Saeed, Christopher N. Selvaraj, Gurkirat Singh, Yogeesan Sivakumaran, Bethany M. Stavert, Elizabeth Suthers, Robert Tang, Vincent C. Varley, Thodur M. Vasudevan, Uyen G. Vo, Timothy Wagner, Judy Wang, Jackie Wong

**Affiliations:** ^1^ Concord Repatriation General Hospital Sydney NSW Australia; ^2^ Western Health Melbourne VIC Australia; ^3^ Royal Perth Hospital Perth WA Australia; ^4^ Royal Melbourne Hospital Melbourne VIC Australia; ^5^ Nepean Hospital Sydney NSW Australia; ^6^ Royal Adelaide Hospital Adelaide SA Australia; ^7^ Vascular and Endovascular Research Network Birmingham UK; ^8^ Port Macquarie Base Hospital Port Macquarie NSW Australia; ^9^ The Alfred Hospital Melbourne VIC Australia; ^10^ Fiona Stanley Hospital Perth WA Australia; ^11^ University Hospital Geelong Geelong VIC Australia; ^12^ Princess Alexandra Hospital Brisbane QLD Australia

**Keywords:** amputation, COVID‐19, frailty, vascular surgery procedures

## Abstract

**Background:**

Frailty predicts adverse perioperative outcomes and increased mortality in patients having vascular surgery. Frailty assessment is a potential tool to inform resource allocation, and shared decision‐making about vascular surgery in the resource constrained COVID‐19 pandemic environment. This cohort study describes the prevalence of frailty in patients having vascular surgery and the association between frailty, mortality and perioperative outcomes.

**Methods:**

The COVID‐19 Vascular Service in Australia (COVER‐AU) prospective cohort study evaluates 30‐day and six‐month outcomes for consecutive patients having vascular surgery in 11 Australian vascular units, March–July 2020. The primary outcome was mortality, with secondary outcomes procedure‐related outcomes and hospital utilization. Frailty was assessed using the nine‐point visual Clinical Frailty Score, scores of 5 or more considered frail.

**Results:**

Of the 917 patients enrolled, 203 were frail (22.1%). The 30 day and 6 month mortality was 2.0% (*n* = 20) and 5.9% (*n* = 35) respectively with no significant difference between frail and non‐frail patients (OR 1.68, 95%CI 0.79–3.54). However, frail patients stayed longer in hospital, had more perioperative complications, and were more likely to be readmitted or have a reoperation when compared to non‐frail patients. At 6 months, frail patients had twice the odds of major amputation compared to non‐frail patients, after adjustment (OR 2.01; 95% CI 1.17–3.78), driven by a high rate of amputation during the period of reduced surgical activity.

**Conclusion:**

Our findings highlight that older, frail patients, experience potentially preventable adverse outcomes and there is a need for targeted interventions to optimize care, especially in times of healthcare stress.

## Graphical abstract

This multicentre cohort study demonstrated that pre‐operative frailty was associated with adverse perioperative outcomes and increased major amputation but not mortality during the early COVID‐19 pandemic. This outcome disparity highlights the importance of targeted interventions to optimise the care of older, frail patients, especially in times of healthcare stress.

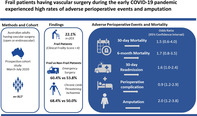

## Introduction

Older patients requiring vascular surgery are at significant risk of adverse outcomes due to higher rates of multimorbidity, frailty and disease severity. The current coronavirus disease 2019 (COVID‐19) pandemic has highlighted the vulnerability of older, frail patients in our healthcare systems. Restrictions in elective surgery, conversion to telehealth^1^ and reduced outpatient services have unmasked key structural and social inequities experienced by older patients accessing surgical care.^2^

Frailty, defined as a multidimensional syndrome or phenotype with reduced physiological robustness,^3^, ^4^ is recognized as an independent prognostic factor for surgical patients with an increased risk of perioperative mortality and morbidity.^5^, ^6^, ^7^ In vascular surgery, frailty is associated with mortality, length of stay, post‐operative complications including infection and readmission, and higher‐level care requirements.^8^, ^9^ Given its predictive association with adverse outcomes after surgery, it is recommended that frailty assessment be included in routine pre‐operative risk stratification to allow early intervention aimed to decreasing geriatric syndromes such as delirium.^10^ Early in the COVID‐19 pandemic responses, frailty was proposed as a potential tool to inform resource allocation and shared decision‐making about surgical care in patients during severe pandemic‐related healthcare constraints.

In the COVID‐19 Vascular sERvice in Australia (COVER‐AU) prospective cohort study, we aimed to establish the prevalence of frailty in patients receiving vascular surgery during the early stages of the COVID‐19 pandemic and investigate the association of frailty with perioperative outcomes of mortality, perioperative complications, and readmissions.

## Methods

### Study design and context

The COVER‐AU study is a multicentre observational cohort study involving 11 vascular surgery units across Australia and lead by the Australian and New Zealand Vascular Trainee Network (ANZVTN), a trainee research collaborative within the Royal Australasian College of Surgeons Clinical Trials Network (CTANZ). All participating trainees received specific training in the study design and data collection. Here, we present the Australian outcomes from the global COVER study designed and initiated by the Vascular and Endovascular Research Network (VERN), the United Kingdom (UK) trainee research collaborative.

### Study participants

Consecutive patients aged 18 years or older, admitted to hospital for vascular surgery between March–July 2020 were prospectively included. Participating units recruited patients over a three‐month period, with start dates varying by approval processes and the local pandemic responses. Participants were excluded if they did not receive a surgical intervention during the study period. Data were collected from 11 participating vascular centres in five states (Appendix;1).

### Data collection

Patient demographics, procedural information and outcome measures were collected at the time of hospital admission, supplemented by review of the medical records and local clinical audit databases. Clinical outcomes were assessed as 30‐days and six‐months follow up, by review of medical records

Frailty was prospectively assessed during hospital admission using the Clinical Frailty Score (CFS), a 9‐point scale based on clinical history and physical examination.^10^, ^11^ The CFS relies on clinician assessment without the use of specific sarcometric measures and has high specificity in vascular patients.^11^, ^12^ Researchers were trained to use the CFS, and provided the visual guide that accompanies the tool.^13^ Patients were allocated a CFS score of 1 ‘Very Fit’, 2 ‘Fit’, 3 ‘Managing Well’, 4 ‘Living with very mild frailty’, 5 ‘Living with mild frailty’, 6 ‘Living with moderate frailty’, 7 ‘Living with severe frailty’, 8 ‘Living with very severe frailty’ or 9 ‘Terminally Ill’.^13^ Patients with a CFS score of 5 or more were considered frail.

All data were collected prospectively into REDCap.^14^, ^15^ Only deidentified data were available for analysis. Because of potential research delays from pandemic‐related disruption, retrospective completion of some data were permitted but all sites maintained a prospective enrolment log.

### Study outcomes

The primary outcome was mortality (in‐hospital, and at 30‐days after hospital admission). Secondary outcomes were perioperative and 30‐day medical and surgical complications, readmissions (within 30‐days after hospital admission) and length of stay (LOS) during the initial vascular surgery admission.

Six‐month outcomes included mortality, and disease specific outcomes such as amputation for peripheral artery disease, and readmission or reintervention within 6 months of initial surgery.

Results were adjusted for confounders of age, and gender, and influence of comorbidity was explored on sensitivity analysis.

### Statistical analysis

Comparative statistics were used to identify associations between outcomes and frailty, with subgroup analysis for specific vascular procedures. Where participant numbers were insufficient for meaningful statistical analysis, descriptive data are presented. Participant characteristics and outcomes were compared using chi‐square test. We used logistic regression to calculate odds ratios (OR) and 95% confidence intervals (CI) for comparative outcomes. Statistical analysis was performed in SAS Ent 1.5 (0.6‐4.0erprise, version 13.1 (SAS Institute Inc., Cary, NC).

Outcomes documented in hospital records were verified by site leads. Where data variables were missing or incomplete, researchers were contacted to provide additional information. After this, participant records with missing CFS or outcome data were excluded from subgroup analyses, specified in the results.

### Ethics

National Human Research Ethics Application approval, including a waiver of consent was granted. Local governance approval was obtained at participating sites. Data‐sharing and collaborative authorship agreements were formalized between the lead Australian site (Sydney Local Area Health Service) and all participating institutions. The Strengthening the Reporting of Observational Studies in Epidemiology (STROBE) guidelines for reporting of cohort studies informed manuscript development.^16^

## Results

**Fig. 1:**
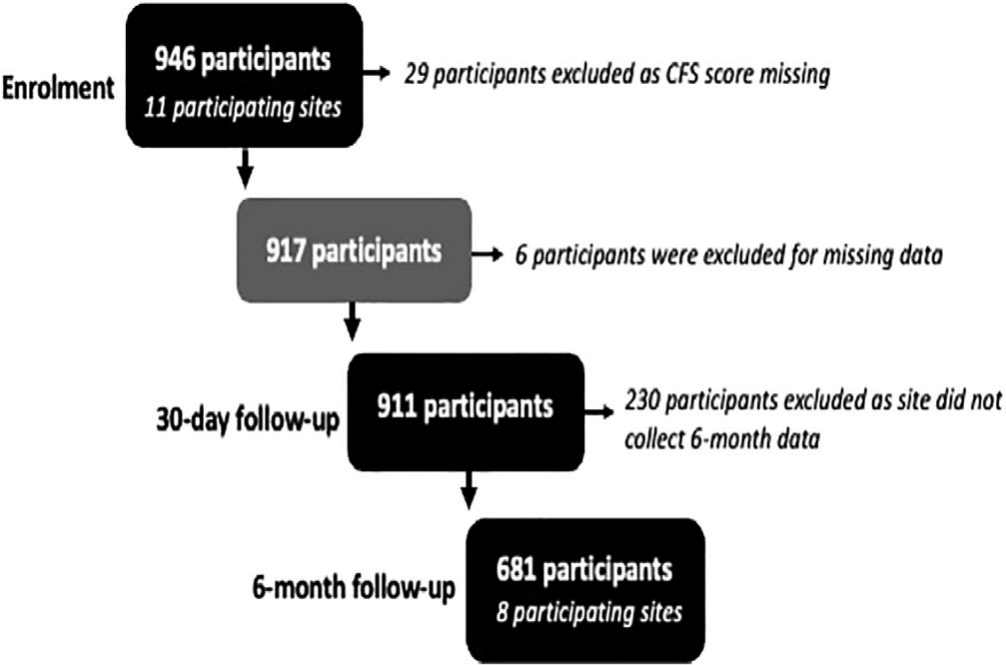
Enrolment and follow up for patients enrolled in the COVER‐AU study. *CFS, Clinical Frailty Score.

Between March and July 2020, 11 vascular units enrolled 946 patients. We excluded 29 participants in whom CFS scores were unable to be obtained. Figure 1 shows complete follow up data was available for 911 participants at 30‐days (99.3%) and for 681 (74.2%) participants at six‐months.

### Patient characteristics

Table 1 shows the participant characteristics. Frailty was present in 203 (22.1%) of patients. Frail patients were older with a mean age of 72.5 ± 12 years compared to 64.8 ± 13 years in non‐frail patients.

**Table 1:** Patient characteristics and operative procedures according to frailty status for patients in Australia having vascular surgery, in the COVID‐19 pandemic from march to July 2020

Characteristics	Frail (CFS 5–9) *n* = 203	Non‐frail (CFS 1–4) *n* = 714	*P*‐value
Female *n*(%)	67 (33.0%)	172 (24.1%)	0.011
Mean age, years (SD)	72.5 ± 12	64.8 ± 13	–
Comorbidities			
Diabetes mellitus	123 (60.6%)	308 (43.1%)	0.001
Hypertension	148 (72.9%)	468 (65.6%)	0.049
Chronic obstructive pulmonary disease	42 (20.7%)	103 (14.4%)	0.031
Ischaemic heart disease	72 (35.5%)	217 (30.4%)	0.170
Chronic kidney disease	68 (33.5%)	129 (18.1%)	<0.001
Stroke/transient ischaemic attack	45 (22.12%)	79 (11.1%)	<0.001
Current smoker	23 (11.3%)	155 (21.7%)	0.001
Cancer	22 (10.8%)	32 (4.5%)	0.001
Dementia	9 (4.4%)	6 (0.8%)	<0.001
Renal replacement Therapy/dialysis	39 (19.2%)	58 (8.1%)	<0.001
SARS‐CoV‐2 infection			
Confirmed	0 (0.0%)	0 (0.0%)	‐
Suspected	1 (0.5%)	1 (0.1%)	‐
Type of operation			<0.001
Carotid endarterectomy	0 (0.0%)	72 (10.1%)	
Lower limb endovascular procedure	46 (22.7%)	178 (24.9%)	
Lower limb open procedure	17 (8.4%)	96 (13.5%)	
Lower limb hybrid procedure	13 (6.4%)	52 (7.3%)	
Amputation or debridement	65 (32.0%)	124 (17.4%)	
Aortic intervention	12 (5.9%)	87 (12.2%)	
Upper limb/thoracic	2 (1.0%)	14 (2.0%)	
Vascular access	46 (22.7%)	82 (11.45%)	
Mesenteric procedure	2 (1.0%)	9 (1.3%)	
Surgical urgency classification			0.106
Elective	81 (39.9%)	330 (46.2%)	
Urgent/emergent	122 (60.1%)	384 (53.8%)	
ASA^†^			<0.001
1	0 (0.0%)	9 (1.3%)	
2	2 (1.0%)	63 (8.8%)	
3	162 (79.8%)	569 (79.7%)	
4	36 (17.7%)	66 (9.2%)	
5	3 (1.9%)	7 (1.0%)	

^†^ American Society of Anaesthestists Score.

Frailty was significantly associated with having more than one comorbidity (OR 7.2, 95% CI 1.7–30.0). Compared to non‐frail patients, the odds of having diabetes mellitus (OR 2.03, 95% CI 1.5, 2.8), chronic kidney disease (OR 2.3, 95% CI 1.6–3.2) and renal replacement therapy (OR 2.7, 95% CI 1.7–4.2) were more than twice as high in frail patients. A greater proportion of non‐frail patients (155, 21.7%) were active smokers, compared to frail patients (23, 11.3%).

### Primary outcomes

Table 2 shows the primary and secondary outcomes. Six of the frail patients died in hospital (3.0%) compared to 12 non‐frail patients (1.7%). A 30‐days follow up, an additional two patients from the non‐frail cohort had died (2.0%) but no further frail patients died. There was no significant difference for in‐hospital (OR 1.8, 95% CI 0.7, 4.8) or 30‐day all‐cause mortality (OR 1.5, 95% CI 0.6–4.0) between frail and non‐frail patients.

**Table 2:** Outcomes of patients having vascular surgery during the march–July 2020 COVID‐19 pandemic in Australian hospitals, according to clinical frailty score at 30‐days follow‐up

30‐day outcomes	Frail (*n* = 203)	Non‐frail (*n* = 714)	Odds ratio^†^ (95% confidence interval)
In‐hospital mortality, *n*(%)	6 (2.96%)	12 (1.68%)	1.8 (0.7–4.8)
30‐day all‐cause mortality, *n*(%)	6 (2.96%)	14 (1.96%)	1.5 (0.6–4.0)
30‐day readmissions, *n*(%)	35 (17.25%)	83 (11.62%)	1.6 (1.03–2.4)
Length of stay, median days (SD)	8 (±13)	5 (±9)	‐
Perioperative complications^‡^			
At least one complication, *n*(%)	38 (18.72%)	78 (10.92%)	1.9 (1.2–2.9)
Myocardial infarction, *n*(%)	4 (1.97%)	13 (1.82%)	1.1 (0.3–3.3)
Pneumonia, *n*(%)	13 (6.40%)	20(2.80%)	2.4 (1.2–4.9)
Surgical site infection, *n*(%)	16 (7.88%)	36 (5.04%)	1.6 (0.9–3.0)
Stent or graft thrombosis, *n*(%)	9 (4.43%)	10 (1.40%)	3.3 (1.3–8.2)
Limb loss^§^, *n*(%)	20 (9.9%)	39 (5.4%)	1.9 (1.1–3.3)

^†^ Unadjusted univariate analysis.

^‡^ Complication occurring in hospital or within 30‐days of discharge.

^§^ Including patients with primary amputation.

### Secondary outcomes

The median length of stay (LOS) was 8 ± 13 days for frail patients compared to 5 ± 9 days for non‐frail patients. On univariable logistic regression analysis, frail patients had almost double the odds of developing at least one complication compared to non‐frail patients. The risk of developing pneumonia was twice as high in frail patients and the risk of graft or stent thrombosis was three times higher in frail patients compared to non‐frail counterparts. 30‐day readmission rate was significantly higher in frail patients compared to non‐frail patients.

### Six‐month outcomes

Table 3 shows the six‐month outcomes. Three of the participating sites were unable to contribute six‐month data, and hence, 230 (25.2%) of 911 patients were excluded from analysis of six‐month outcomes. Complete six‐month follow up was available for 681 patients, 154 of whom were frail (22.6%). Overall, the all‐cause mortality at 6 months was 5.9%, with 27 non‐frail and 13 frail patients dying. After adjusting for age and sex, frailty was not associated with increased mortality risk in patients for whom 6 month follow up was available (OR 1.7, 95%CI 0.8–3.5). The risk of readmission, and reoperation were significantly higher in patients who were frail compared to those who were not, after adjusting for age and sex.

**Table 3:** Outcomes of patients having vascular surgery during the early COVID‐19 pandemic in Australian hospitals, according to frailty status at 6 months follow up

Six‐month outcomes	Frail (*n* = 154)	Non‐frail (*n* = 527)	Odds ratio^†^ (95% confidence interval)
All‐cause mortality, *n* (%)	12 (7.8)	23 (4.4)	1.68 (0.79–3.54)
Reoperation, *n* (%)	53 (34.4)	137 (26.0)	1.67 (1.12–2.49)
Readmission, *n* (%)	67 (43.5)	189 (35.9)	1.51 (1.03–2.20)
Amputation, *n* (%)	21 (13.6)	39 (7.4)	2.01 (1.17–3.78)

^†^ Adjusted for age and sex.

Frail patients were at twice the odds compared to non‐frail patients to receive an amputation during their initial hospital admission or in the subsequent 6 months (OR 2.0, 95%CI 1.2–3.8) after adjusting for age and sex.

### Subgroup analysis of specific procedures

The most frequent procedure was lower limb revascularisation (402, 44%), either open, endovascular or hybrid. Frail patients were more likely to have an endovascular procedure (41, 54.0% versus 160, 49.1%), or need urgent lower limb revascularisation (47, 61.8% versus 160, 49.1%) compared to non‐frail patients. The odds of chronic‐limb threatening ischaemia in frail patients were twice that of non‐frail patients (52, 68.4% versus 162, 50.0%; OR 2.2, 95% CI 1.3–3.7), with frail patients more likely to have tissue loss than rest pain compared to non‐frail patients (OR 3.0, 95% CI 1.8–5.0). Major amputation during initial vascular admission was performed for 20 (9.9%) frail patients and 39 (5.4%) non‐frail patients, with frail patients having a higher rate of failed revascularisation in the 14 days prior to their amputation.

Aortic intervention was performed on 12 (5.9%) frail and 87 (12.2%) non‐frail patients, most frequently endovascular. Ruptured aneurysm repair occurred in four frail patients (33.3%) and eight non‐frail patients (9.2%). Urgent aortic intervention was performed for seven frail patients (57.3%) compared to 42 non‐frail patients (48.3%). The main indication for surgery was aneurysmal disease above the size threshold and frail patients had a larger mean aortic diameter than non‐frail (63 mm versus 59 mm).

No frail patients received carotid endarterectomy during the study period, but 72 non‐frail patients had carotid surgery (10.1%), with the majority for symptomatic carotid disease (53, 73.6%).

Vascular access operations were performed in 82 (11.5%) non‐frail patients and 46 (22.6%) frail patients, with arteriovenous fistula creation the most frequent procedure. Surgical revision of existing vascular access was more commonly performed in non‐frail patients, while endovascular revision was more commonly in frail patients.

## Discussion

Our study demonstrates that frailty is common in patients admitted to hospital for vascular surgery, with a prevalence of 22.1% for all ages, increasing to 28.4% of patients aged 65 years or older. This prevalence was similar to other Australian cohort studies, conducted prior to the COVID‐19 pandemic. McRae *et al*. found 40% of patients aged 65 years or older having vascular surgery in a Brisbane tertiary hospital were frail.^17^ A more recent study identified frailty in 30% of patients aged 65 years or older admitted to the vascular service in a Sydney tertiary hospital.^18^ Our findings show that frailty was associated with worse perioperative outcomes after vascular surgery, but not mortality. Frail patients averaged an extra 3 days in hospital, were more likely to have at least one complication by the time of discharge, and more likely to be readmitted to hospital within a month of surgery, compared to non‐frail patients. This placed considerable demand on healthcare systems already strained by the pandemic response.

Within our cohort, the vascular disease profile, and operative interventions performed on frail patients differed to patients who were not frail. The reduced surgical capacity may have shifted the complexity of the vascular casemix to include more frail patients than pre‐pandemic ratios, as frail patients are likely to present emergently with complex, time sensitive vascular diseases that require life or limb saving procedures.^19^ Despite this, outcomes for lower limb intervention in frail patients were comparable to pre‐pandemic outcomes.^20^, ^21^, ^22^, ^23^ For instance, frail patients were more likely to receive intervention for chronic limb‐threatening ischaemia than non‐frail patients. As a result, they were also more likely to require a major amputation compared to non‐frail patients. Frail patients were also more likely to undergo urgent or emergent lower limb intervention. However, frail patients were less likely to receive procedures aimed at preventing a future adverse disease state, such as surgery for asymptomatic carotid disease or aortic aneurysm. Our study design does not allow for deeper exploration of the clinical decision making behind these differences in treatment. One factor influencing decision making could be the higher rates of adverse operative outcomes occurring in frail patients‐ for example there was a higher rate of early graft thrombosis in frail patients compared to those who were not. Patient treatment preferences, long term survival and anticipated functional gains may also influence treatment options provided to frail patients.

Our findings demonstrate the Australian all‐cause mortality 30‐days after vascular surgery was low at 2.0%. and 5.9% at 6‐months. This contrasts with the global COVER cohort study, where 30‐day all‐cause mortality occurred in 9.0% of participants, much higher than pre‐pandemic vascular cohort studies.^24^ No patients in our cohort had surgery whilst infected with SARS‐CoV‐2, and only one subsequently tested positive, in contrast to the global results where 15 (1.4%) patients were COVID‐19 positive.^24^, ^25^ In Australia, the initial wave of the pandemic was considerably smaller than seen in countries such as the United Kingdom and the United States, with the healthcare demand largely managed by restricting hospital services to emergent care.^26^, ^27^ This was in contrast with the large first wave experienced in countries such as the UK where hospital services were overwhelmed leading to significant changes in practice^25^ and may contribute to the differences observed between the Australian results and the global COVER outcomes.

As Australian surgical services experience ongoing COVID‐19 related disruption with the Omicron variant, the lessons from the initial responses are important to draw on. Frailty was used as a screening tool for limiting hospitalization of older patients in the UK during periods of heightened pandemic‐related hospital strain, resulting in considerable community and consumer groups backlash due to the potential for ageist discrimination.^28^ Acute hospital care is not always futile or burdensome for older frail patients, and can result in significant improvements in survival, quality of life and function. Our study findings demonstrated clinical benefit was gained by providing vascular care for frail, older patients in our cohort, with an overall low morbidity and longer‐term survival gains. Rather than viewing frailty assessment as a method for restricting care, it can be used to identify groups of patients who would benefit from more targeted, nuanced therapy. Models of care where geriatricians collaborate with vascular surgeons have resulted in decreased hospital acquired complications and geriatric syndromes when used during the COVID‐19 pandemic.^29^ Frailty assessment can enhance shared decision‐making conversations with patients and family about prognosis, emphasizing aspects of care that relate to physical function and independence, advanced care directives and quality of life.

Our study is strengthened by its multicentre, prospective design and high participant numbers. To our knowledge, this study is the largest prospective Australian trial examining the impact of frailty on vascular surgery patients both before and during the COVID‐19 pandemic. Interobserver variability was reduced by using a validated frailty score with a robust training method for clinician researchers who were actively involved in direct and consecutive patient care. Another strength was the prospective evaluation of real‐time clinical adaptations and models of care in response the pandemic changes in hospital service. Whilst the multicentre recruitment provides a representative sample of vascular practice in Australia, this represented only one fifth of vascular training units and may underestimate variation in both COVID‐19 disruption and regional practice. A key limitation is the low incidence of the primary mortality outcomes, underpowering our analysis despite high enrolment and limiting the value of subgroup analyses. Due to some sites not participating in long‐term follow up, complete data at 6 months was missing for a quarter of the cohort, increasing the risk of selection bias influencing the outcomes. It is also possible that some outcome variables were not collected if patients were subsequently treated at non‐participating vascular units, although this risk was reduced by centralisation of vascular services and shared electronic health records. Subgroup analysis showed that the characteristics and procedural distribution remained proportional between groups, but this low rate of follow‐up limits the reliability of long‐term outcomes.

## Conclusion

This multicentre cohort study demonstrated that pre‐operative frailty was associated with adverse perioperative outcomes but not mortality during the early COVID‐19 pandemic. This outcome disparity highlights the importance of targeted interventions to optimize the care of older, frail patients, especially in times of healthcare stress. Future longitudinal research should be undertaken to examine the impact of frailty on vascular surgery in a post‐pandemic setting.

## Conflict of interest

The authors have no financial or personal conflicts of interest to declare.

## Funding information

No funding was received for conducting this study or in the preparation of this manuscript. The authors declare they have no financial interests.

## Appendix 1 Participating hospitals and authors

### 1.1

Participating Hospitals

NSW: Concord Repatriation General [lead site], Nepean, Port Macquarie,

VIC: The Alfred, Royal Melbourne, University Hospital Geelong, Western Health,

SA: Royal Adelaide,

WA: Royal Perth, Fiona Stanley,

QLD: Princess Alexandra.

Authors

Writing and Analysis Team.

Kai Wen Leong, Jana‐Lee Moss, and Sarah Joy Aitken.

Vascular and Endovascular Research Network Study Leads

Ruth Benson and Sandip Nandhara.

Data Collection and Australian Study Implementation

Aitken SJ, Khoo S, Charlton G, Mehta S, Lim E, Papanikolas M, Stavert B, Vasudevan T, Varley V, Dean A, Coleman C, Ravintharan N, Fitridge R, Dawson J, Kuang B, Hon K, Chambers J, Gan J, Suthers E, Selvaraj C, McClure D, Wang J, Kilby J, Allard B, Pond F, Saeed H, Liu JWN, Tang R, Aziz O, Singh G, Richards T, Moss J, Vo UG, Dhal BS, Atkinson N, Wagner T, Musicki K, Leong KW, Hattam A, Muller J, Sivakumaran Y, Altaf N, Wong J, Thurston B, Picazo F, Hanna J, Hine M.
